# Benchmarking vector arthropod culture: an example using the African malaria mosquito, *Anopheles gambiae* (Diptera: Culicidae)

**DOI:** 10.1186/s12936-016-1288-4

**Published:** 2016-05-10

**Authors:** Laura Valerio, C. Matilda Collins, Rosemary Susan Lees, Mark Q. Benedict

**Affiliations:** Department of Experimental Medicine, University of Perugia, Perugia, Italy; Centre for Environmental Policy, Imperial College London, Silwood Park, Ascot, UK; Polo d’Innovazione Genomica, Genetica e Biologia S.C.a.R.L., Edificio D, 3º piano Polo Unico di Medicina ´Santa Maria della Misericordia´, Loc. S. Andrea delle Fratte, 06132 Perugia, Italy; Insect Pest Control Section, Joint FAO/IAEA Division of Nuclear Techniques in Food and Agriculture, Department of Nuclear Applications, International Atomic Energy Agency, Vienna International Centre, PO Box 100, 1400 Vienna, Austria; Centers for Disease Control and Prevention (CDC), 1600 Clifton Road, Atlanta, GA 30329 USA

**Keywords:** Insect culture, Standard operating procedure, Vector capacity, Insecticide resistance, Bioassay, Diet, Morphometrics

## Abstract

**Background:**

Numerous important characteristics of adult arthropods are related to their size; this is influenced by conditions experienced as immatures. Arthropods cultured in the laboratory for research, or mass-reared for novel control methods, must therefore be of a standard size range and known quality so that results are reproducible.

**Methods:**

A simple two-step technique to assess laboratory culture methods was demonstrated using the mosquito *Anopheles gambiae s.s.* as a model. First, the ranges of key development outcomes were determined using various diet levels. The observed outcomes described the physiologically constrained limits. Secondly, the same outcomes were measured when using a standard operating procedure (SOP) for comparison with the determined ranges.

**Results:**

The standard method resulted in similar development rates to those of high and medium diets, wing length between those resulting from the high and medium diets, and larval survival exceeding all benchmark diet level values. The SOP used to produce experimental material was shown to produces high-quality material, relative to the biologically constrained limits.

**Conclusions:**

The comparison between all possible phenotypic outcomes, as determined by biological constraints, with those outcomes obtained using a given rearing protocol is termed “benchmarking”. A method is here demonstrated which could be easily adapted to other arthropods, to objectively assess important characters obtained, and methods used, during routine culture that may affect outcomes of research.

**Electronic supplementary material:**

The online version of this article (doi:10.1186/s12936-016-1288-4) contains supplementary material, which is available to authorized users.

## Background

The global public health significance of mosquitoes is manifested by continuing high mortality and morbidity rates due especially to dengue and malaria. Their public health importance results in them being a common subject of research, usually for the purpose of improving or developing control methods, including insecticides, repellents, genetic and biological methods. In addition, they are used similarly to a reagent for evaluation of disease control approaches that do not control the mosquito directly such as vaccines and drugs.

In the absence of reproducible laboratory mosquito culture, unnecessary and undesirable variability in the data collected due to variation in mosquito characteristics is likely to be introduced. For example, one easily measured characteristic, size, has been reported to affect numerous traits relevant to disease transmission, including host seeking and repellence by DEET [1], susceptibility to arboviruses [2], longevity [3], dispersal [4], and fecundity [5]. It is well established that larval culture is the strongest determinant of adult size, fecundity and longevity [6–8]. The weight of diet available/larva is particularly important [8].

Regardless of the known importance of size, in the absence of a comprehensive published literature or agreed guidelines, methods for culturing mosquitoes and other vector arthropods often rely on *ad hoc* personal judgement and experience to determine the larval density and amount of food required for acceptable development and survival rates. Characteristics used to judge culture conditions for mosquitoes often use water qualities such as opacity, colour or smell in conjunction with the larval stage, density and perception of mosquito vigour to judge the amount of food provided. This is often referred to as ad libitum, although in fact, when provided in such a way, diet may not be available in sufficient amounts for unlimited feeding.

Although culturing by an experienced and conscientious person can produce predictable outcomes, an alternative is to systematize the culture methods, thus creating a more portable technique that does not depend upon the skill of an individual to such an extent. A standard operating procedure (SOP) that specifies the diet to be used, the amounts, the larval density, containers, amount and type of water, and temperature provides such a method. Accurate use of SOPs results in reproducible outcomes and possibly standardizes outcomes of various research activities. Methods for consistent production of mosquitoes have been reported, usually in the context of mass production, where rearing must necessarily be systemized (for examples see [9, 10]).

Consistency, however, does not provide any measure of the intrinsic quality of mosquitoes. The same mosquito strains consistently produced within various laboratories may differ greatly among them. An objective external standard of quality would allow assessment and comparison of methods used to culture mosquitoes in different laboratories. One such method is to compare laboratory-cultured mosquitoes with the wild-type population. For example, the size of mosquitoes destined for field release has been compared with those collected from the field [11–13].

A systematic method to assess a rearing procedure, and to standardize arthropod culture methods between facilities aiming to rear the same strain of arthropods with the same diet and obtain comparable results, would be a valuable addition to these efforts. A simple procedure is therefore proposed to assess insect culture, particularly for mosquitoes: benchmarking. The benchmarking method implicitly reflects the assumption that, under fixed extrinsic conditions such as a particular diet, temperature and density, there are genetically and physiologically determined limits to growth rate and size and where survival is assumed to be determined largely by the specific characteristics of the culture methods but not by intrinsic species characteristics.

As a demonstration application, an SOP that has previously used for production of *Anopheles gambiae* was examined. As the external benchmark, a reproducible method was applied for exploring the genetically constrained factors: development rate and wing length. Survival was also measured from egg hatch to the pupal stage. This is recommended as a method for determining quality that can be adapted to any mosquito species and many other insects.

## Methods

Insectaries where mosquitoes were held averaged 27.6 °C (±0.01), 81.9 % RH (±0.09), (95 % CIs of the mean). A 12:12 h light: dark schedule was employed with a 30 min simulated dawn and dusk. On the day of hatching, 16 first instar larvae (L1s) of the G3 strain (obtained from the Malaria Research and Reference Reagent Resource Center) were counted into standard polystyrene 90-mm Petri dishes containing 30 ml of a standard rearing water (Milli-Q, Integral Water Purification System, Darnstadt, Germany) containing 0.3 g/l artificial pond salts (Tonic Pond Salts, Aquatics, UK). Each was given 640 μl of either 0.5, 1.0 or 2.0 %w/v diet containing 2:2:1 by weight tuna meal (Progressive Baiting, Dietenhofen, Germany), liver powder (Now Foods, Bloomingdale, IL, USA) and Vanderzant vitamin mix (BioServ, Frenchtown, NJ, USA) [14] in Milli-Q water. Dishes were given 640 μl of diet on alternate days. Diet concentrations were chosen based on previous experience as providing low, medium and high levels for *Anopheles arabiensis* [8] (Table 1). Four dishes of each combination of larval concentration and diet were established.

**Table 1:** Culture parameters of the experiments

Treatment	Starting number (L1s)	Liquid Volume (ml)	Density (larvae /ml)	Feeding schedule	Diet volume (ml)	Diet concentration % w/v
Low diet	128	30	4.3	Alternate days	0.64	0.5
Medium diet	128	30	4.3	Alternate days	0.64	1.0
High diet	128	30	4.3	Alternate days	0.64	2.0
SOP	750	175	4.3	According to the SOP	According to the SOP	2.0

* Feeding regime according to the SOP (Additional file 1) as follows - Days 1 and 3: 5ml, Day 4: 7ml, Day 5: 10ml, Days 6 and 7: 10-12ml, thereafter: by judgement

Pupae were collected daily, their sex determined by examination of terminalia and then they were pooled by diet level and transferred to insectary cages. Adults were provided with a solution of 10 % sucrose and 0.1 % (both w/v) methylparaben in Milli-Q water. Dead adults were stored, the right wing was removed when not damaged, or alternatively the left wing, and dry mounted on a microscope slide using double-stick transparent adhesive tape. Wing length was measured as the distance from the axial incision to the R4+5 vein excluding the fringe seta using ImageJ software [15].

The SOP is provided as Additional file 1. Briefly, it uses the same diet and a similar number of larvae/ml of water as the Petri dish experiments, one larva/2 ml water. Larval development time was measured from the day of hatching to the day of pupation. The SOP feeding schedule uses 2 %w/v of the same diet as used for the Petri dishes, but is fed in volumes which increase, beginning with 5 ml on day 0 (hatching at a density up to 1000 L1/500 ml in a 35 × 25 × 8-cm plastic tray), 5 ml on day 1 when the larvae are reduced in density to 250/tray, and then given 0, 5, 7, 10, and 10–12 ml on days 3–7, respectively. On day 7 and thereafter, the amount of food is adjusted according to a judgement of the number of larvae remaining (Table 1).

All statistical analyses were performed using R 3.0.1 [16]. Analysis of variance was used for the wing length data to assess the influence of main effects (diet level, sex, experiment) and their interactions. The significance of all terms was assessed by deletion testing from an initial maximal model. Larval duration (the number of days from hatch to pupation) was measured for all individuals reaching pupation. These measures were nested in the dish from which they came, and to avoid pseudoreplication a mixed-effects model using the R package ‘lme4’ was used to account for the random effect of ‘dish’. The proportion of each replicate (dish) pupating was analysed by quasi-binomial general linear model (GLM) to account for the overdispersion found in these data.

## Results

**Fig. 1:**
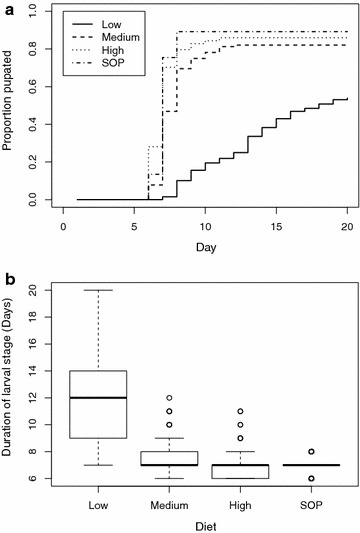
**a** Proportion of starting numbers of larvae that pupated by day for each treatment and **b** duration of the larval stage by treatment. Heavy *black lines* represent the median, the *boxes* are the interquartile range, the whiskers the extent of data unless outliers (*circles*) more than 1.5 times the interquartile range from the median are present

The proportions of L1s reaching the pupal stage differed with diet level (Table 2, Fig. 1a, [17]). The proportions of pupae obtained with the SOP did not differ from that of either high (F = 0.45, df = 1,23, p = 0.51) or medium (F = 1.9, df = 1,23, p = 0.17) diets and these did not differ from each other (F = 0.29, df = 1,23, p = 0.58). The low diet level resulted in the lowest pupal production relative to the other three combined (F = 20.5, df = 2,24, p < 0.001). Pupation when using the SOP usually commenced on day 7.

**Table 2:** Survival of larvae to the pupal stage when cultured under four different conditions

Treatment	Number of experiments	Number of dishes	Starting number (L1s)	Number pupating	Proportion surviving	Standard deviation
Low diet	2	8	128	64	0.500	0.192
Medium diet	2	8	128	105	0.820	0.140
High diet	2	8	128	110	0.859	0.145
SOP	1	3	750	669	0.892	0.032

Similar trends were observed in the duration of the larval stage (Fig. 1b, [17]). There was variation in larval duration as a function of diet (L ratio = 40.2, df = 3,6, p < 0.001): the SOP and the high level diet did not differ (L ratio = 1.7, df = 5,6, p = 0.18), nor was the medium diet different from these (L ratio = 2.2, df = 4,5, p = 0.13). Only the low level diet resulted in a longer larval stage (L ratio = 36.2, df = 3,4, p < 0.001).

Wing length was used as an indicator of adult body size. Wing length was continuous and normally distributed. The explanatory variables were categorical: the sex of the mosquito, the experimental runs and the four diet levels (high, medium, low and SOP). Analysis of variance was appropriate and a maximal model was fit to the data to assess the influence of main effects and their interactions. Initially, the maximal model included both sexes, but as male and female wing length are known to differ, a fact confirmed by these data, the sex-specific responses to diet and experiment were examined separately.

The experimental-runs vector was partially confounded with diet levels as the wing measurements for the low, medium and high diet came from two separate experimental runs and those of SOP from standard culturing. A separate ANOVA asked whether the wing length differed between experimental runs. It did not (F = 2.04, df = 1291, p = 0.15) and this factor was excluded from subsequent analyses.

**Fig. 2:**
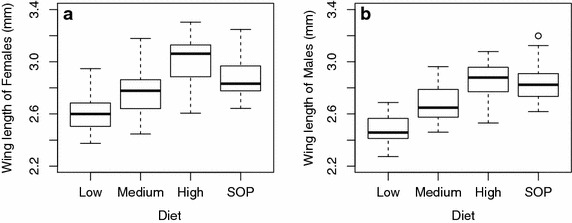
Wing length of mosquitoes as a function of treatment: three diet levels in Petri dishes and SOP culturing. **a** Female mosquitoes; **b** male mosquitoes

There was a significant interaction between diet level and sex (F = 2.7, df = 286,289, p < 0.05) indicating that the effect varied between males and females. Males responded to, but were less responsive to, the high diet than were females (Fig. 2).

For females there was an overall effect of larval culture, and wing length increased with diet quantity (F = 35.4, df = 3143, p < 0.001). Individuals fed on the low diet had shorter wings than those fed on a medium diet (F = 11.2, df = 1143, p < 0.01), and the high diet led to longer wings than medium (F = 49.3, df = 1143, p < 0.001). The SOP feeding regime gave rise to wing lengths between those of the medium and high diet but distinct from both (SOP: medium, F = 13.4, df = 1143, p < 0.001, SOP: high, F = 18.5, df = 1143, p < 0.001) (Fig. 2a, [17]).

Male wing length also varied with diet level (F = 39.1, df = 3143, p < 0.001). Males fed the medium diet had longer wings than those fed the low diet (F = 23.3, df = 1143, p < 0.001), and those fed the high diet had longer wings still (F = 25.8, df = 1143, p < 0.001). The SOP gave rise to wing lengths greater than those of the medium diet (F = 28.5, df = 1143, p < 0.001) and indistinguishable from the high diet (F = 0.67, df = 1143, p = 0.41) (Fig. 2b, [17]).

## Discussion

The simple method that is described here provided a benchmark for an SOP that was developed independently for the culture of *An. gambiae*. The same method can be applied to other mosquitoes and insects that are cultured under controlled conditions and that can be manipulated to affect their development rate and size. Other outcomes such as adult emergence, fecundity, longevity, or mating rates could be used as benchmark characters depending on the interests of the programme. The Petri dish observations could in themselves be used as the basis for developing an SOP, though that was not done in this case, however, preliminary observations demonstrated that in most regards, scaling the Petri dish density and diet levels up to routinely used tray size and larval number resulted in a similar outcome as was obtained in the Petri dishes. In a small set of experiments to test this, the same feeding schedule for the low, medium and high diet levels was increased proportionally and used to rear 250 L1s in the rearing trays described in the Methods. This resulted in higher survival in the medium and high diet trays than that obtained in the equivalent Petri dish experiments, but lower survival at the low level (survival to pupation was 43.9, 90.8 and 86.7 % in low, medium and high diet treatments, respectively). The wing length of females (mean = 2.88 μ, n = 16, std. 95 % CI = ± 0.04) and males (mean = 2.73 μ, n = 18, 95 % CI = ± 0.04) cultured at the high diet level was similar to that of the SOP samples.

Differences, if supported by further experiments, illustrate that the conditions experienced in small containers differ in some way not explained simply by larval and food density. The amount of food provided in the SOP corresponds to providing 3.8 mg of food per larva during the first seven days, as compared to the 3.2 mg per larva provided during the same period in the high diet Petri dishes. Even though the SOP provided more total diet during the first seven days than the high-level Petri dishes, the females produced were smaller, though female size was more consistent under the SOP conditions, possibly due to the more uniform conditions resulting from increased scale. Even considering that the diet amounts reported in Methods were based on the starting number of larvae rather than those that actually survived to pupation, which differed between the high and SOP experiments, the amount of food provided was still higher (4.2 and 3.7 mg/pupa for the SOP and high Petri dish, respectively). Thus, the reduced female size in the SOP experiment may reflect an effect of the relatively smaller amount of food provided early in development. Possibly, the larvae cultured under the SOP would have benefitted from a larger proportion of the total food upon hatching or on day 1 than was provided. This notion, that not simply the total amount of diet but the timing of its provision affects development, may be supported by the preliminary observations made when scaling up the Petri dish experiments to trays.

**Fig. 3:**
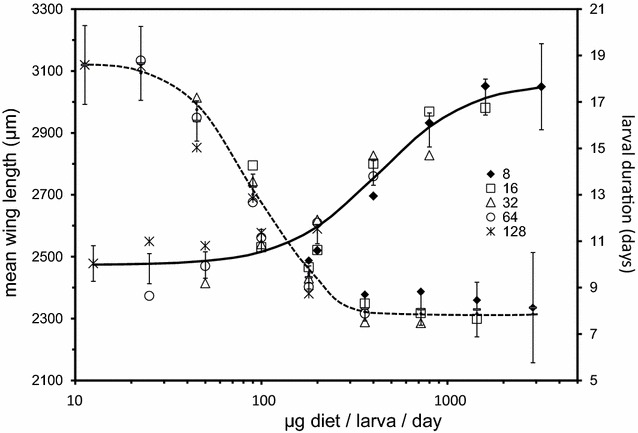
Relationship of larval duration to wing length ([8], Fig. 3). The best fit of larval duration (descending dashed line, values on right y-axis) and wing length (ascending solid line, left y-axis) are plotted as a function of μg diiet/llarva/d. Individual points are mean wing lengths and larval durations calculated as means of replicates by experiment at each larval density/diet amount. Error bars are 95% confidence intervals of the mean. Diet/initial number larva/d predicted larval duration and wing length with good certainty (R2 = 0.932 and R2 = 0.975, respectively). Values that describe this curve as in the equation described in the text for the minimum, difference in minimum and maximum, slope, and X midpoint for wing length were 2,473.12, 590.71, 0.423, and 1.7718. The values for larval duration were 7.86, 10.91, −2.2305, and 0.095

Using development rate as an outcome in the absence of wing length or some measure of size for mosquitoes is not advised. In nature, larvae may develop in temporary water sources under conditions of limited nutrition, leading to the evolution of strategies to balance increased size and shorter development time, a trade-off which is likely sex-specific. The relationship between different developmental parameters is, therefore, not always linear (discussed in detail in [18]). Gilles et al. ([8] Fig. 3) demonstrated that larval development rates are maximal at diet levels above which significant increases in wing length are still possible. Therefore, development rate alone could be maximized under conditions that still gave significantly smaller adults than could be obtained.

The method used here does not provide an absolute benchmark. It is possible that other diets or culture conditions could produce a wider range of outcomes, and variation between rearing conditions in different facilities will alter the results. For example, Kivuyo et al. [19] generally observed larval durations much longer than those observed in these benchmarking experiments, and a much greater disparity in pupation rates, when testing new larval diets for *An. gambiae*. This could be explained by the different temperature and relative humidity in which the experiments were conducted. In contrast, similar feeding regimes have led to a similar range of male wing lengths in different hands [20]. Phelan and Rotiberg [18] recorded very similar values for these two parameters to the current study, in an experiment to determine the effects of food availability, water depth and temperature, despite the differences in culture methods and diet used. The mean days to pupation reported here for the medium, high and SOP experiments are similar to that of the best diet used at the lowest larval concentration by Kivuyo et al. [19] and Damiens et al. [14] and Gilles et al. [8] for *An. arabiensis*, seven to eight days. Similarly, the wing lengths observed at the medium and high levels were similar to those observed using the same diet by Damiens et al. [14] who reported means of 2903 and 2783 μ for females and males, respectively.

When alternative diets or culture conditions are tested, they can in turn be benchmarked using the method described here and used to expand the array of measured responses. Providing a fixed amount of diet over the course of larval development may be more effective, or different larval densities may result in faster development or larger size. In contrast, the specific diet used was developed for culture of the *An.gambiae* sibling species, *An. arabiensis*, by an optimization protocol [8] and is believed to be of high quality, so further increases in development rate, survival and size in this case may be difficult to achieve by using a different diet alone.

## Conclusion

By culturing insects under a range of conditions that achieve the intrinsic upper and lower limits of development rate and size, one can determine this range for a given diet, temperature and strain. By comparing the outcomes from an SOP with the full range of measured parameters, an estimate of the quality of the insects produced by the SOP can be determined. It would be useful for a rearing or research facility to benchmark their SOP using a given strain, diet and temperature. Culture methods could thus be objectively and quantitatively evaluated and compared.

## Additional file

10.1186/s12936-016-1288-4 Standard Operating Procedure for standardised routine culture of *Anopheles gambiae*, developed and employedby Polo d’Innovazione Genomica, Genetica e Biologia S.C.a.R.L., Perugia, Italy.

## Abbreviations

GLM: general linear model
L1s: first instar larvae
SOP: standard operating procedure
