# Supplementary material for: Benchmarking vector arthropod culture: an example using the African malaria mosquito, Anopheles gambiae (Diptera: Culicidae)
Source: Malar J. 2016 May 10;15:262. doi: 10.1186/s12936-016-1288-4 (PMC4862070; doi:10.1186/s12936-016-1288-4)

# Culturing larvae

## Brief description of the purpose

The protocol is designed to achieve similar high outcomes of *Anopheles gambiae* survival, adult size and development rate in mosquito rearing. Following the protocol is expected to produce mosquitoes that can be compared to one another at any given time with some assurance that they are similar.

## Background

Mosquito larval culture is typically septic, and the diet consists of both added food and the microbial growth that results. Reliable diets provide good nutrition directly, are easy to purchase and use. Studies have shown that above a minimum weight threshold, the number of eggs laid per female is in fairly direct proportion to their adult weight. Adult size is ultimately determined by larval size, and that depends on larval culture. So, it is logical that larger larvae yield higher numbers of eggs per female – a desirable outcome in most laboratory cultures. Consistent use of successful practices developed and proven in each lab should be adhered to faithfully.

When larval culture fails, laboratorians often suspect something is wrong with the water. In spite of this concern, many different sources of water have been used successfully - in combination with good rearing techniques: chlorinated/fluoridated municipal supply, deionized, untreated deep well, and distilled water. Water source is probably not a critical factor for most species unless it contains high levels of toxic chemicals. If in doubt, try changing to a more purified form in controlled experiments (i.e. side-by-side with the same cohort of eggs) until suitable conditions are found. The consistency of the source should be considered in making the choice as this will affect long-term success.

Some chemical treatment methods used in municipal supplies may not be compatible with larval mosquitoes. Chloramine is reportedly of particular concern for invertebrate culture. The rule of thumb is to test the water source with a small number of larvae before using it on a larger scale. If you are concerned about chlorine residues, use water from a hot water source that is allowed to cool, or chlorinated water that is allowed to sit overnight. For critical applications where physiologically uniform water is required, we use reverse/osmosis deionized UV sterilized water to which we add 0.3 g / liter artificial sea salts such as Instant Ocean™.

Water that is mixed with salt in large volumes should not be left in the same container indefinitely as a biofilm will form. Rotate the container weekly and allow the empty one to dry thoroughly in a dry place – not in an environmental chamber.

A water temperature of 26.5-28°C is suitable for rearing most anophelines. The room or incubator temperature should be adjusted to achieve this depending on whether the trays are covered or not. Uncovered trays will have a water temperature significantly below the air temperature due to evaporative cooling. Trays placed high in a room will often be at temperatures significantly above that of lower ones if the air does not circulate well. This should be checked.

Diet is provided as a 2% weight/volume slurry prepared in purified water. The slurry must be well mixed before every pipetting. Only a volume of diet should be prepared as can be used in about 3 days to prevent spoilage. Keep the diet in the refrigerator when not in use, and keep on ice if it must be kept out for periods longer than about 20 minutes.

In Perugia insectary we use a diet made of bovine liver powder, tuna meal and Vanderzant vitamin mix in a 2:2:1 ratio (Damiens et al., 2012). See the protocol “Preparing Damiens et al. larval diet in bulk” for its preparation.

## Materials:

| 1 liter of 2% w/v larval diet  10 ml disposable pipet  pipet aid  purified water  mosquito rearing water (0.3 g/liter sea salts in purified water)  mosquito trays  laminated schedules (at bottom)  china marker (grease pencil) | plexiglass tray covers  fabric tray covers (optional)  90 mm plastic Petri dishes  eggs on damp filter paper  rearing water  wash bottle containing water  label tape  permanent fine marker |
| --- | --- |

As you follow the protocol, mark the date and initial each activity on the laminated schedule sheets on the day it is performed.

| **Day** | **Activity** | **Notes** |
| --- | --- | --- |
| 0 | Bleach eggs and store overnight on damp filter paper. |  |
| 1 | 1. Place tray containing 500 ml rearing water and 5 ml of diet on a rearing shelf. 2. Without moving the tray, wash up to 1000 eggs into the tray. If more larvae will be needed, hatch in multiple trays, each containing < 1000 eggs. 3. Place a new label on each tray with the stock name, generation or cross and date. If multiple hatching trays are set up, attach an identical label to each additional tray. 4. Tape the laminated schedule sheet to the tray, date and initial it. Keep with cohort. | Tray should be covered. |
| 2 | 1. Concentrate the larvae with a fine strainer and re-suspend in 100 ml of rearing water. 2. Pour the larvae into a 90 mm plastic Petri dish until there are approximately 250 in the dish. This will be a density in the range between the 200 and 300 photographs, but attempt to match the 250 larvae image. 3. Transfer the contents of each dish to a tray containing 500 ml of rearing H_2_O and 5 ml of food. 4. Label each tray with stock name, generation and date of hatching. 5. Repeat until the desired number of trays has been set up. | Keep the laminated schedule sheet attached to one of the trays in the cohort. |
| 3 | Add no food |  |
| 4 | 5 ml food |  |
| 5 | 7 ml food | Trays may remain uncovered. |
| 6 | 10 ml food |  |
| 7 & + | 10-12 ml food depending on water clarity – 12 if clear, 10 if cloudy | Pupae should begin forming. Combine trays to maintain ~ 250 larvae per tray. |

## Supporting Documentation

Methods in Anopheles Research, Second Edition (2011) available at:

<http://www.mr4.org/AnophelesProgram/TrainingMethods.aspx>

Damiens D, Benedict MQ, Wille M, Gilles JRL 2012. An inexpensive and effective larval diet for Anopheles arabiensis (Diptera: Culicidae): eat like a horse, a bird, or a fish? J. Med. Entomol. 49(5): 1001-1011


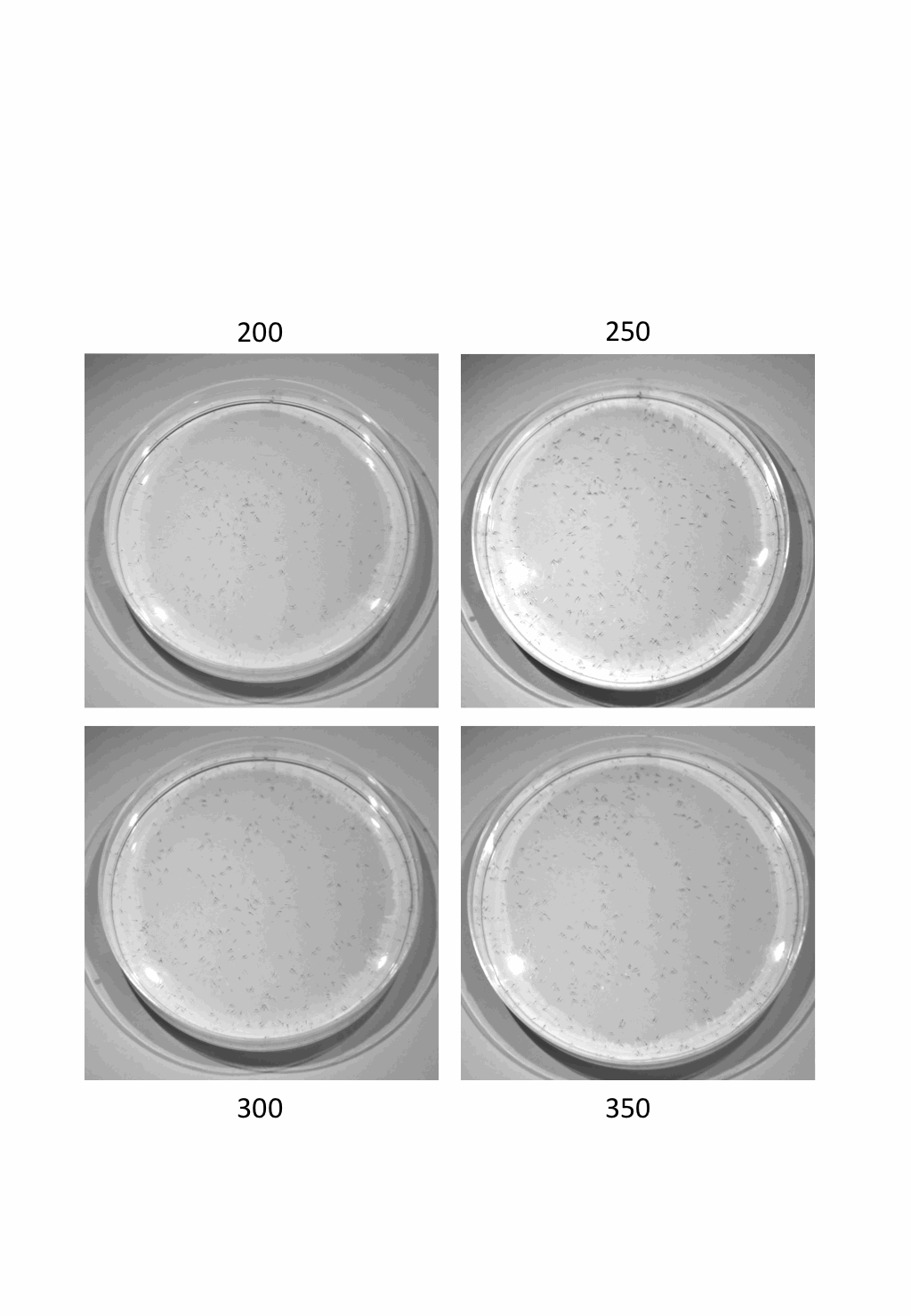

Supplement: Supplementary file 1 — 10.1186/s12936-016-1288-4 Standard Operating Procedure for standardised routine culture of Anopheles gambiae, developed and employedby Polo d’Innovazione Genomica, Genetica e Biologia S.C.a.R.L., Perugia, Italy. [file 12936_2016_1288_MOESM1_ESM.docx]
